# FHIR PIT: an open software application for spatiotemporal integration of clinical data and environmental exposures data

**DOI:** 10.1186/s12911-020-1056-9

**Published:** 2020-03-11

**Authors:** Hao Xu, Steven Cox, Lisa Stillwell, Emily Pfaff, James Champion, Stanley C. Ahalt, Karamarie Fecho

**Affiliations:** 1 Renaissance Computing Institute, University of North Carolina at Chapel Hill, Chapel Hill, North Carolina 27517 USA; 2 North Carolina Translational and Clinical Sciences Institute, University of North Carolina at Chapel Hill, Chapel Hill, North Carolina 27599 USA

**Keywords:** Data integration, Spatiotemporal data, Modular software design

## Abstract

**Background:**

Informatics tools to support the integration and subsequent interrogation of spatiotemporal data such as clinical data and environmental exposures data are lacking. Such tools are needed to support research in environmental health and any biomedical field that is challenged by the need for integrated spatiotemporal data to examine individual-level determinants of health and disease.

**Results:**

We have developed an open-source software application—FHIR PIT (Health Level 7 Fast Healthcare Interoperability Resources Patient data Integration Tool)—to enable studies on the impact of individual-level environmental exposures on health and disease. FHIR PIT was motivated by the need to integrate patient data derived from our institution’s clinical warehouse with a variety of public data sources on environmental exposures and then openly expose the data via ICEES (Integrated Clinical and Environmental Exposures Service). FHIR PIT consists of transformation steps or building blocks that can be chained together to form a transformation and integration workflow. Several transformation steps are generic and thus can be reused. As such, new types of data can be incorporated into the modular FHIR PIT pipeline by simply reusing generic steps or adding new ones. We validated FHIR PIT in the context of a driving use case designed to investigate the impact of airborne pollutant exposures on asthma. Specifically, we replicated published findings demonstrating racial disparities in the impact of airborne pollutants on asthma exacerbations.

**Conclusions:**

While FHIR PIT was developed to support our driving use case on asthma, the software can be used to integrate any type and number of spatiotemporal data sources at a level of granularity that enables individual-level study. We expect FHIR PIT to facilitate research in environmental health and numerous other biomedical disciplines.

## Background

Researchers and healthcare practitioners across fields of biomedicine acknowledge the tremendous impact that environmental exposures have on health and disease. For example, airborne pollutant exposures have been linked to diseases as diverse as asthma [1–6], diabetes [7–9], cardiovascular disease [10], dementia [11], mental health disorders [12], obesity [13], liver disease [14], and premature mortality [15]. Yet, informatics tools to study the interaction between environmental exposures and health outcomes at the level of the individual are largely non-existent. For instance, the fields of epidemiology and environmental health focus primarily on population-based correlations between trends in spatiotemporal exposures and population-level health outcomes [15]. Longitudinal clinical studies likewise are limited in their ability to collect subject-level data on environmental exposures, typically relying on survey-based self-report [5] or expensive personal monitors [6]. Electronic health record (EHR)–based research also is limited because such records do not contain data on environmental exposures apart from basic demographics.

Herein, we present FHIR PIT (Health Level 7 Fast Healthcare Interoperability Resources Patient data Integration Tool) as an open-source software application designed to overcome challenges in environmental health research and related fields and provide an innovative solution to enable investigation into the impact of individual-level environmental exposures on health and disease.

## Implementation

This work was conducted under a study protocol that was approved by the Institutional Review Board at the University of North Carolina at Chapel Hill.

### Motivation

FHIR PIT is a complex, custom, open-source software application that uses geocodes and time stamps of varying resolution (e.g., hour, day, year) to automatically integrate multiple sources of spatiotemporal data, irrespective of the degree to which the data depend on space and time. FHIR PIT was motivated by our research and development of the Integrated Clinical and Environmental Exposures Service [ICEES [16]. ICEES was developed as part of the Biomedical Data Translator program in response to a need to openly expose clinical data that have been integrated at the patient and visit level with environmental exposures data [17, 18]. FHIR PIT provides the integrated clinical and environmental exposures data to support ICEES.

### Implementation overview and spatiotemporal data sources

**Fig. 1:**
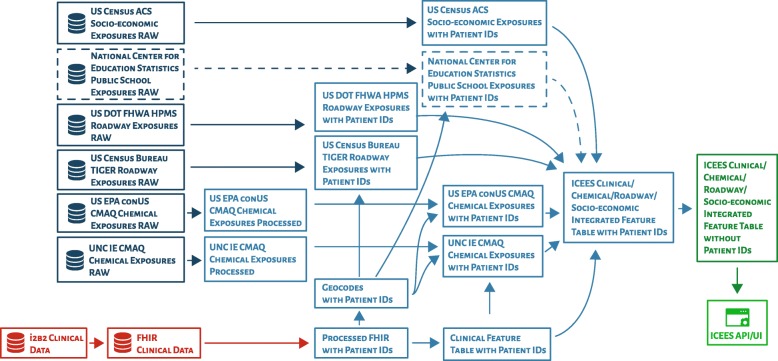
An overview of the integration steps embedded in the FHIR PIT software application pipeline. API = application programming interface; FHIR = Health Level 7 Fast Healthcare Interoperability Resources; ICEES = Integrated Clinical and Environmental Exposures Service; UI = user interface; US Census ACS = US Census Bureau’s American Community Survey; US Census Bureau TIGER = US Census Bureau’s Topologically Integrated Geographic Encoding and Referencing system; US EPA conUS CMAQ = US Environmental Protection Agency’s conUS Community Multiscale Air Quality modeling data; US DOT FHWA HPMS = US Department of Transportation, Federal Highway Administration, Highway Patrol Monitoring System. Red color = sensitive, fully identified clinical data; dark blue color = public data on environmental exposures; light blue color = secure, firewall- and Institutional Review Board–protected integration steps; dark green color = de-identified, binned integrated feature tables; light green color = ICEES OpenAPI. (*Note that data from the National Center for Education Statistics have not yet been integrated using FHIR PIT, but an approach is under development to integrate data on school exposures with home exposures data and clinical data, thereby addressing issues related to patient mobility and differential exposures. A simplified version of the FHIR PIT pipeline was published in* JAMIA *2019;26(1):1064–1073 and is reprinted in adapted form here with full permission from the publisher. In contrast to the simplified version of the FHIR PIT pipeline, the version shown here includes and clearly distinguishes all of the data sources and integration steps that are assembled by the current version of the pipeline.)*

For initial research and development of FHIR PIT, clinical data on patients from UNC Health Care System were integrated with a variety of public data on environmental exposures, including: airborne pollutant exposures from the US Environmental Protection Agency; roadway exposures from the Federal Highway Administration’s Highway Patrol Monitoring System, within the US Department of Transportation; roadway exposures from the US Census Bureau’s Topologically Integrated Geographic Encoding and Referencing system; and socio-environmental exposures from the US Census Bureau’s American Community Survey. (A graphical overview of the FHIR PIT integration pipeline can be found in Fig. 1. A list of currently available feature variables can be found in Supplementary Table 1. This table and additional documentation are maintained and regularly updated on the ICEES OpenAPI.) Importantly, the integration step is conducted within a secure environment and under a protocol that was approved by our institution’s Institutional Review Board because data integration necessitates the use of patient geocodes (i.e., primary home residence), date/time stamps, and patient identifiers—data elements that are considered Protected Health Information under the Health Insurance Portability and Accountability Act (HIPAA).

Multiple integration steps are required to harmonize across these data sources, which vary in spatiotemporal resolution and format of geocodes and time stamps. For example, patient primary home residence is coded as latitude and longitude in the patient data, whereas the American Community Survey data are provided at the Census block level. Airborne pollutant exposures are available at hourly estimates, daily estimates, or annual averages, depending on the exposure entity and source year. Roadway data are provided as GIS shape files, with latitudes and longitudes in WGS84 decimal format, which is the World Geodetic System for expressing latitude and longitude. Separate software code is required to convert the spatiotemporal representation of the data used by each data source into a common format that allows integration across data sources. In addition, separate mappings are required to link patient identifiers and geocodes with each non-clinical data source, thereby supporting the final integration step that merges the different data sources.

The final product of the FHIR PIT software pipeline is a set of “integrated feature tables”, with feature variables binned or recoded and data de-identified according to §164.514(b) of HIPAA for subsequent open access via the ICEES OpenAPI.

### Implementation details

FHIR PIT consists of several transformation steps or building blocks that can be chained together to form a transformation and integration workflow. Several of these transformation steps are generic, such that they can take in any data that conform to a certain format. Thus, the incorporation of new types of data amounts to adding new transformation steps or reusing generic steps. FHIR PIT is implemented using Apache Spark. Spark is used to easily parallelize and distribute the data transformation steps. A Python script is used to simplify the application interface to the transformation steps. FHIR PIT supports building containers in both Singularity and Docker. This feature allows the application to run on different machines and platforms with portability.

Each block in FHIR PIT is implemented as a plugin consisting of a set of Scala classes that can be plugged into the pipeline. FHIR PIT is configured using a YAML file, and steps can be switched on or off for rapid re-execution of the pipeline. The plugins consist of both generic building blocks such as joining of tables and data set–specific building blocks such as preprocessing of environmental data (Table 1). The input and output of each plugin can be configured so that the output of the previous step in a pipeline configuration can be fed as input for the next step.

**Table 1:** FHIR PIT plugin names and functionalities

Plugin name	Functionality
FHIR	Consolidates different FHIR resources for each patient and extracts geocodes
ToVector	Extracts features from FHIR
EnvData	Preprocesses environmental data source
CSVTable	Converts to ICEES integrated feature table
ACS	Preprocesses US Census Bureau ACS data source
ACS2	Preprocesses US Census Bureau ACS data source, v2; this includes a “ur” field for “urban or rural” residence
NearestRoad	Preprocesses nearest road data source for US Census Bureau TIGER data source
NearestRoad2	Preprocesses nearest road data source for US DOT FHWA HPMS data source
NOOP	No operation

One of our goals for implementation of the pipeline is to enable automatic and rapid re-execution. Given the extensible number of input files and parameters, we use the Dhall configuration language to author configuration files and avoid code duplication. Dhall code is converted to a YAML file that is then read by the pipeline. An example YAML configuration of a step in the FHIR PIT pipeline is provided below, with fields defined in Table 2.

**Table 2:** FHIR PIT field names and functionality

Field name	Functionality
name	Designates name of given step instance
dependsOn	Defines other step instances that given step instance depends on
skip	Determines whether given step instance should be skipped; if skip is “true”, then this step will not be run; skip function allows for partial re-execution of pipelines that have not been completely executed
step	Defines the given step instance
step.function	Designates the function name for given step instance; this is usually a class name
step.arguments	Delineates specific arguments for given step function; the arguments vary according to the step function

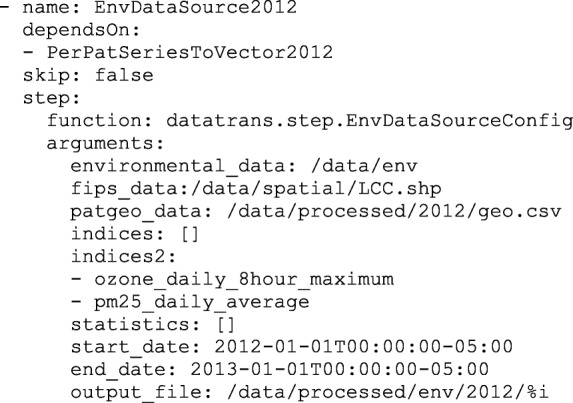



Writing the entire FHIR PIT pipeline configuration in YAML would necessitate rewriting the pipeline for every new calendar year and every new data set. With Dhall, we are able to create a function in the configuration that can be instantiated for each new calendar year or data set. A simplified version of this function to address additional years is shown below.

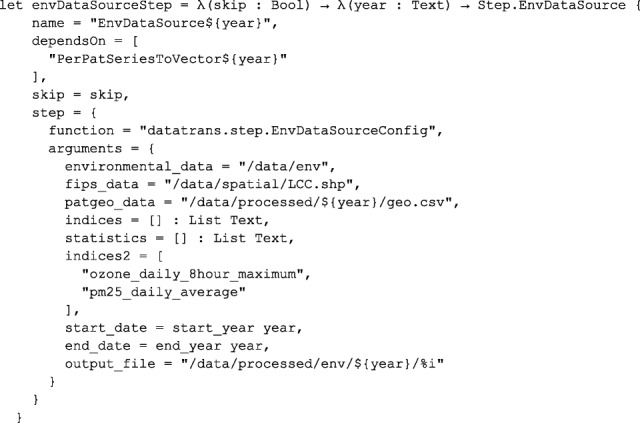



To instantiate this for calendar year 2012, we simply need to specify the following parameter:

envDataSourceStep False "2012"

To extend this function for multiple calendar years, we specify an additional parameter:

List/map ["2012", "2013", "2014"] (envDataSourceStep False)

Here, the *List/map* function takes a list of terms and a function, applies the function to each element in the list, and returns a list of values.

Execution of the FHIR PIT pipeline generates a report of skipped tasks, succeeded tasks, failed tasks, and errors from failed tasks.

## Results

We validated FHIR PIT in the context of our driving use case for research and development of ICEES: impact of airborne pollutant exposures on asthma. The validation data set consisted of ~ 160,000 patients with “asthma-like” conditions from UNC Health Care System and the environmental data sources depicted in Fig. 1, focusing initially on data from calendar year 2010 [19–21]. FHIR PIT was used to integrate the clinical and environmental data and then de-identify the data and bin feature variables before openly exposing the integrated data using ICEES. ICEES was queried using the following input parameters:

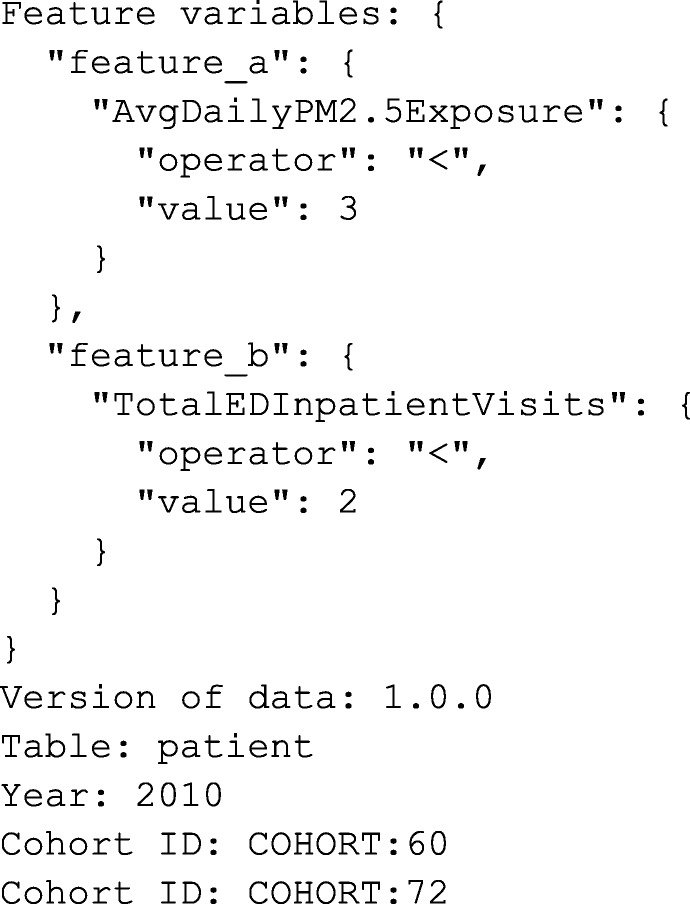



**Fig. 2:**
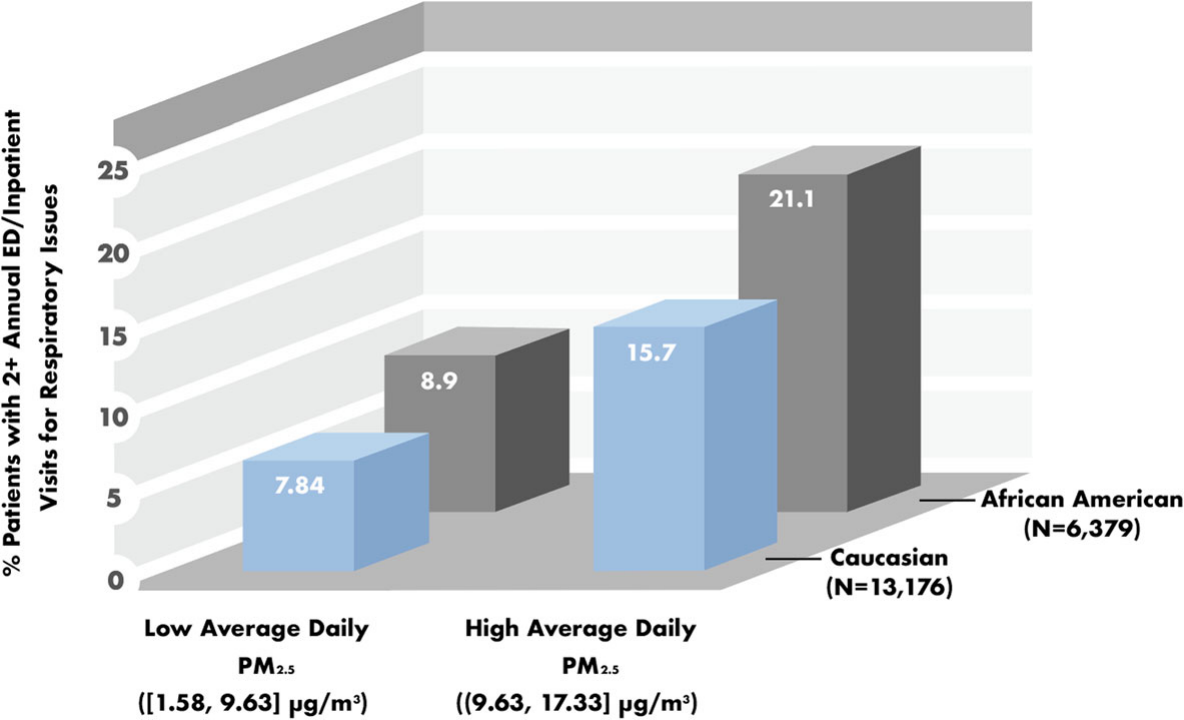
Racial disparities in the impact of airborne pollutant exposures on asthma exacerbations. Sample sizes are: *N* = 6379 African American patients; and *N* = 13,176 Caucasian patients. PM_2.5_ = particulate matter < 2.5-μm in diameter. Levels of PM_2.5_ exposure were binned in FHIR PIT using pandas qcut and expressed as ranges. *X*^2^ = 28.2841, *P* < 0.0001 for African Americans; *X*^2^ = 47.0133, *P* < 0.0001 for Caucasians

ICEES returned the following JSON output, which is also displayed in graphical form in Fig. 2.

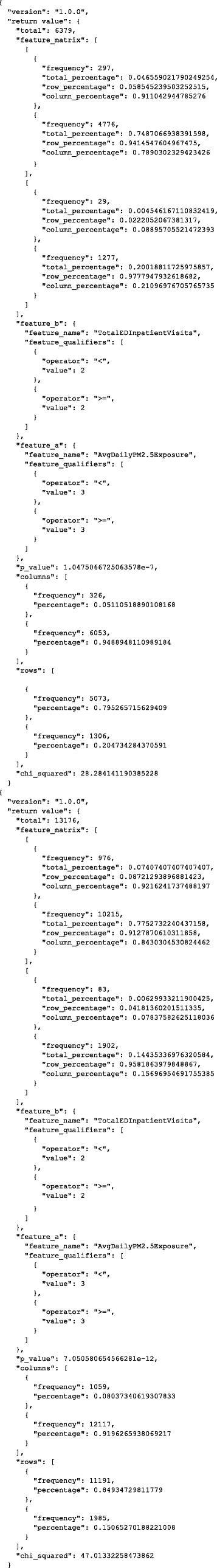



These results indicate that the proportion of patients with two or more annual emergency department or inpatient visits for respiratory issues was higher among patients exposed to relatively high average daily levels of particulate matter < 2.5-μm in diameter (PM_2.5_) than among those exposed to relatively low average daily levels of PM_2.5_. Moreover, asthma exacerbations, as defined by two or more annual emergency department or inpatient visits for respiratory issues, were more common among African Americans than among Caucasians.

We then examined prednisone use in relation to asthma exacerbations among African Americans and Caucasians. The ICEES query is shown below.

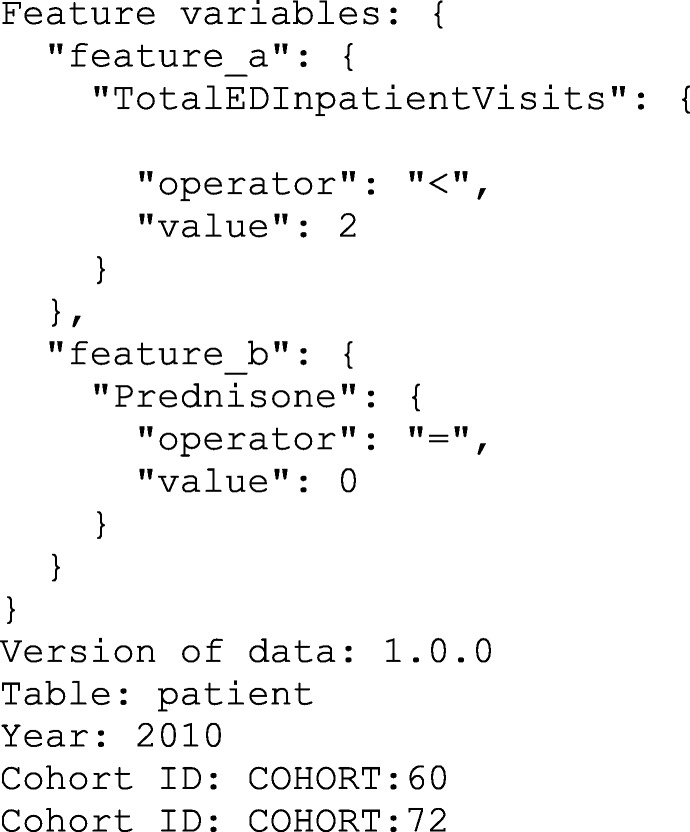



ICEES returned the following results, which are shown in tabular form in Table 3.

**Table 3:** Relationship between prednisone use and asthma exacerbations, defined as two or more annual ED or inpatient visits for respiratory issues, among African Americans and Caucasians

	Patients with < 2 annual ED/inpatient visits for respiratory issues N (%)	Patients with ≥ 2 annual ED/inpatient visits for respiratory issues N (%)	Chi square, ***P*** value
**African Americans (N = 6379)**			
*Prednisone*			
No	4536 (89.41%)	1078 (82.54%)	*X*^*2*^ = 46.4781,
Yes	537 (10.59%)	228 (17.46%)	*P* < 0.0001
**Caucasians (N = 13,176)**			
*Prednisone*			
No	10,071 (89.99%)	1675 (84.38%)	*X*^*2*^ = 54.8241,
Yes	1120 (10.01%)	310 (15.62%)	*P* < 0.0001

*Abbreviations: ED, emergency department*

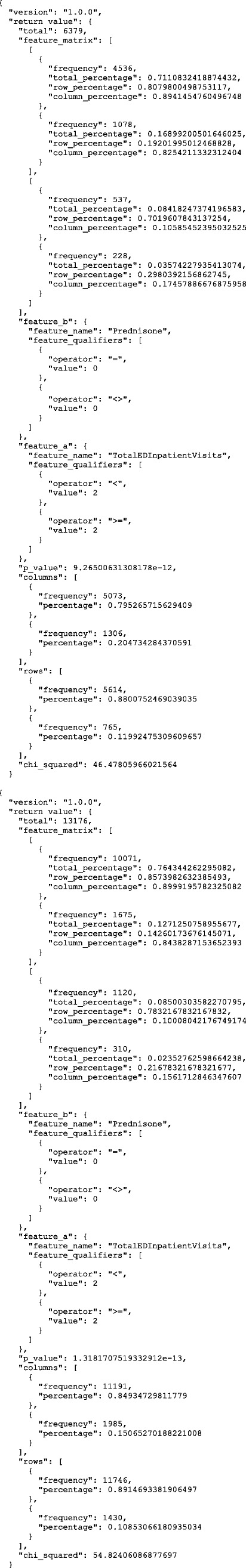



These results indicate that prednisone use was more common among patients with asthma exacerbations than among those without asthma exacerbations, as expected given that prednisone is generally reserved for patients with severe disease [22]. While this finding was true for both African Americans and Caucasian, the effect was more pronounced among African Americans than among Caucasians.

In sum, we successfully applied FHIR PIT to integrate clinical and environmental data and then openly expose the data for interrogation via ICEES, thereby replicating and extending published literature demonstrating the impact of exposure to airborne particulate matter on asthma (e.g., 4) and the existence of racial disparities in asthma exacerbations [23].

## Conclusion

We developed FHIR PIT as an open-source spatiotemporal data integration tool. We are currently using FHIR PIT to generate integrated clinical and environmental data for open exposure and interrogation via ICEES. While FHIR PIT was developed and validated in the context of a driving use case designed to evaluate the impact of airborne pollutant exposures on asthma, the software application has broad applicability in any use case that requires integrated spatiotemporal data for individual-level analysis. Indeed, we are currently extending FHIR PIT to support investigations into the impact of environmental exposures on primary ciliary dyskinesia, drug-induced liver injury, and several additional conditions. We believe that FHIR PIT will facilitate research in environmental health and many other biomedical disciplines.

FHIR PIT is under active development, with new data types and sources planned for the use cases noted above and others. The modular design of FHIR PIT will allow us to rapidly adapt the pipeline for these new data types and sources and automatically execute the pipeline to generate new ICEES integrated feature tables, thus providing flexibility and extensibility. These features will facilitate the adoption and adaptation of FHIR PIT for use in other applications and at other institutions.

## Availability and requirements

- Project name: FHIR PIT
- Project home page: Software code and instructions for downloading FHIR PIT can be found at: https://github.com/NCATS-Tangerine/FHIR-PIT
- Operating system(s): Linux
- Programming language: Scala, Python
- Other requirements: Java 8 or higher
- License: MIT
- Any restrictions to use by non-academics: none

## Supplementary information

**Additional file 1: Supplementary Table 1.** ICEES integrated feature variable tables (v1.0.0, v2.0.0): variable names, descriptions, and binning strategy.*

## Data Availability

The data that were used to validate FHIR PIT are openly available via ICEES at https://icees.renci.org/apidocs.

## Abbreviations

API: Application programming interface
ED: Emergency department
FHIR: HL7 Health Level 7 Healthcare Interoperability Resources
FHIR PIT: Health Level 7 Fast Healthcare Interoperability Resources Patient data Integration Tool
ICEES: Integrated Clinical and Environmental Exposures Service
PM_2.5_: particulate matter < 2.5-μm in diameter
UI: User interface
US Census ACS: US Census Bureau’s American Community Survey
US Census Bureau TIGER: US Census Bureau’s Topologically Integrated Geographic Encoding and Referencing system
US DOT FHWA HPMS: US Department of Transportation, Federal Highway Administration, Highway Patrol Monitoring System
US EPA conUS CMAQ: US Environmental Protection Agency’s conUS Community Multiscale Air Quality modeling data
