# Supplementary material for: FHIR PIT: an open software application for spatiotemporal integration of clinical data and environmental exposures data
Source: BMC Med Inform Decis Mak. 2020 Mar 11;20:53. doi: 10.1186/s12911-020-1056-9 (PMC7066811; doi:10.1186/s12911-020-1056-9)
Supplement: Supplementary file 1 — Additional file 1: Supplementary Table 1. ICEES integrated feature variable tables (v1.0.0, v2.0.0): variable names, descriptions, and binning strategy.* [file 12911_2020_1056_MOESM1_ESM.docx]

## *Supplementary Material*

## **FHIR PIT: an open software application for spatiotemporal integration of clinical data and environmental exposures data**

Hao Xu,^1^ Steven Cox,^1^ Lisa Stillwell,^1^ Emily Pfaff,^2^ James Champion,^2^ Stanley C. Ahalt,^1^ Karamarie Fecho^1^

^1^Renaissance Computing Institute, University of North Carolina at Chapel Hill, Chapel Hill, North Carolina, USA 27517

^2^North Carolina Translational and Clinical Sciences Institute, University of North Carolina at Chapel Hill, Chapel Hill, North Carolina, USA 27599

**Supplementary Table 1.** ICEES integrated feature variable tables (v1.0.0, v2.0.0): variable name, description, and binning strategy.*

| **Feature Variable** | **Description and binning strategy** |
| --- | --- |
| CohortID | *Cohort ID# & input variables used to define cohort* |
| PatientID | *Randomly assigned patient identifier* |
| StudyPeriod | *Years 2010, 2011, 2012, 2013, 2014, 2015, 2016* |
| ***CDWH Clinical Data (years 2010-2016)*** | |
| AgeStudyStart | *Age at ‘study’ start date: calculated from birth date and binned as 0...2, 3...17, 18...34, 35...50, 51...69, 70–89 years*^†^ |
| Sex | *Male, Female, Unknown, Other* |
| Sex2 | *Male, Female* |
| Race | *Caucasian, African American, Asian, Native Hawaiian/Pacific Islander, American/Alaskan Native, Other* |
| Ethnicity | *Hispanic (1=Yes, 0=No)* |
| ObesityDx | *ICD code†† for obesity anytime over 'study' period (1=Yes, 0=No)* |
| ObesityBMI | *BMI ≥ 30 anytime over 'study' period (1=Yes, 0=No)* |
| Diagnoses:  Asthma and asthma-like diagnoses:  AsthmaDx  CroupDx  ReactiveAirwayDx  CoughDx  PneumoniaDx  Diagnoses with known or suspected sex differences:  UterineCancerDx  CervicalCancerDx  OvarianCancerDx  ProstateCancerDx  TesticularCancerDx  KidneyCancerDx  PregnancyDx  MenopauseDx  EndometriosisDx  OvarianDysfunctionDx  TesticularDysfunctionDx  DiabetesDx  AlopeciaDx  FibromyalgiaDx  AlcoholDependenceDx  DrugDependenceDx  DepressionDx  AnxietyDx  AutismDx | *One or more diagnoses for select conditions (see list in left column) (defined by high-level ICD categories) over 'study' period; 1=Yes, 0=No)* |
| Medications Prescribed or  Administered:  Medications for asthma-like conditions:  Prednisone  Fluticasone  Mometasone  Budesonide  Beclomethasone  Ciclesonide  Flunisolide  Albuterol  Metaproterenol  Diphenhydramine  Fexofenadine  Cetirizine  Ipratropium  Salmeterol  Arformoterol  Formoterol  Indacaterol  Theophylline  Omalizumab  Mepolizumab | *One or more prescriptions/administrations of select medications (see list in left column) over 'study' period (1=Yes, 0=No)* |
| ***US Census Bureau ACS Data (years 2007-2011 & 2012-2016 survey samples)*** | |
| EstResidentialDensity | *US Census Bureau ACS 2012-2016 estimated total population [block group], binned according to US Census Bureau definitions (1=rural [0,2500), 2=urban cluster [2500,50000), 3=urbanized area [50000,inf))* |
| ur | *US Census Bureau ACS 2012-2016 estimated urban or rural residence, binned according to US Census Bureau definitions* |
| EstResidentialDensity25Plus  **2012-2016 sample only* | *US Census Bureau ACS 2012-2016 estimated total population aged 25 years or older [block group], binned as quintiles (pandas qcut) (1, 2, 3, 4, 5)* |
| EstPropNonHispWhite | *US Census Bureau ACS 2012-2016 estimated proportion of persons who are non-Hispanic white [block group], binned as quartiles (pandas qcut) (1, 2, 3, 4)* |
| EstPropHouseholdNonHispWhite | *US Census Bureau ACS 2012-2016 estimated proportion of households that are non-Hispanic white [block group], binned as quartiles (pandas qcut) (1, 2, 3, 4)* |
| EstPropHighSchoolMaxEducation | *US Census Bureau ACS 2012-2016 estimated proportion persons aged 25 y or older with a HS diploma or less at their highest level of schooling [block group], binned as quartiles (pandas qcut) (1, 2, 3, 4)* |
| EstPropHighSchoolDropout | *US Census Bureau ACS 2012-2016 estimated proportion persons aged 16-19 y who are neither attending school nor HS graduates [block group], binned as quartiles (pandas qcut) (1, 2, 3, 4)* |
| EstPropHighSchoolDropoutNoWork | *US Census Bureau ACS 2012-2016 estimated proportion persons aged 16-19 y who are neither attending school nor HS graduates and are without work [block group], binned as quartiles (pandas qcut) (1, 2, 3, 4)* |
| EstPropHouseholdsNoAuto | *US Census Bureau ACS 2012-2016 estimated proportion of households without an automobile [block group], binned as quartiles (pandas qcut) (1, 2, 3, 4)* |
| EstPropHouseholdsNoHealthIns  **2012-2016 sample only* | *US Census Bureau ACS 2012-2016 estimated proportion of persons without health insurance [block group], binned as quartiles (pandas qcut) (1, 2, 3, 4)* |
| EstProp5PlusESL | *US Census Bureau ACS 2012-2016 estimated proportion of persons 5 years or older who sometimes speak a language other than English at home [block group], binned as quartiles (pandas qcut) (1, 2, 3, 4)* |
| EstMedianHouseholdIncome | *US Census Bureau ACS 2012-2016 estimated median household income [block group], binned as quintiles (pandas qcut) (1, 2, 3, 4, 5)* |
| EstPropMaleLittleWork | *US Census Bureau ACS 2012-2016 estimated proportion of males aged 16-64 years who worked less than 26 weeks in previous year [block group], binned as quintiles (pandas qcut) (1, 2, 3, 4, 5)* |
| EstPropHouseholdSSI | *US Census Bureau ACS 2012-2016 estimated proportion of households receiving Supplemental Security Income block group], binned as quintiles (pandas qcut) (1, 2, 3, 4, 5)* |
| EstPropHouseholdPA | *US Census Bureau ACS 2012-2016 estimated proportion of households receiving Public Assistance [block group], binned as quintiles (pandas qcut) (1, 2, 3, 4, 5)* |
| EstPropFemaleHouseholdNoSpouse | *US Census Bureau ACS 2012-2016 estimated proportion of family households headed by a female (no male partner present) [block group], binned as quintiles (pandas qcut) (1, 2, 3, 4, 5)* |
| EstPropFemaleHouseholdFamilyChild | *US Census Bureau ACS 2012-2016 estimated proportion of total households headed by a female with family children aged 18 y or less (no male partner present) [block group], binned as quintiles (pandas qcut) (1, 2, 3, 4, 5)* |
| EstPropFemaleHouseholdAnyChild | *US Census Bureau ACS 2012-2016 estimated proportion of total households headed by a female with any children aged 18 y or less (no male partner present) [block group], binned as quintiles (1, 2, 3, 4, 5)* |
| ***US Census TIGERline Roadway Data (year 2016)*** | |
| MajorRoadwayHighwayExposure | *US Census TIGERline distance in meters from household to nearest major road/highway (1 = 0-49, 2 = 50-99, 3 = 100-199, 4 = 200-299, 5 = 300-499, 6 = >=500 meters)* |
| UNC IE 2010 & 2011 CMAQ exposure estimates: PM2.5, ozone^§^  Variable nomenclature format:  AvgDaily**XX**Exposure_StudyAvg  MaxDaily**XX**Exposure_StudyAvg  AvgDaily**XX**Exposure_StudyMax  MaxDaily**XX**Exposure_StudyMax  AvgDaily**XX**Exposure_StudyAvg_qcut  MaxDaily**XX**Exposure_StudyAvg_qcut  AvgDaily**XX**Exposure_StudyMax_qcut  MaxDaily**XX**Exposure_StudyMax_qcut | *UNC IE average of estimated average daily PM2.5 exposure over 'study' period, binned by data values (pandas cut) (1, 2, 3, 4, 5)*  *UNC IE average of estimated maximum daily exposure over 'study' period, binned by data values (pandas cut) (1, 2, 3, 4, 5)*  *UNC IE maximum of estimated average daily PM2.5 exposure over 'study' period, binned by data values (pandas cut) (1, 2, 3, 4, 5)*  *UNC IE maximum of estimated maximum daily exposure over 'study' period, binned by data values (pandas cut) (1, 2, 3, 4, 5)*  *UNC IE average of estimated average daily PM2.5 exposure over 'study' period, binned by quintiles (pandas qcut) (1, 2, 3, 4, 5)*  *UNC IE average of estimated maximum daily exposure over 'study' period, binned by quintiles (pandas qcut) (1, 2, 3, 4, 5)*  *UNC IE maximum of estimated average daily PM2.5 exposure over 'study' period, binned by quintiles (pandas qcut) (1, 2, 3, 4, 5)*  *UNC IE maximum of estimated maximum daily exposure over 'study' period, binned by quintiles (pandas qcut) (1, 2, 3, 4, 5)* |
| US EPA 2002-2016 conUS CMAQ exposure estimates: PM2.5, ozone **  Variable nomenclature format:  AvgDaily**XX**Exposure_2  MaxDaily**XX**Exposure_2  AvgDaily**XX**Exposure_2_qcut  MaxDaily**XX**Exposure_2_qcut | *US EPA conUS CMAQ daily exposure estimates for PM2.5, CO, NO, NO2, NOx, SO2, acetaldehyde, formaldehyde, or benzene, averaged over ‘study’ period, binned by data values (pandas cut) (1,2,3,4,5)*  *US EPA conUS CMAQ 8-hour ozone maximum exposure estimate, averaged over 'study' period, binned by data values (pandas cut) (1, 2, 3, 4, 5)*  *US EPA conUS CMAQ daily exposure estimates for PM2.5, CO, NO, NO2, NOx, SO2, acetaldehyde, formaldehyde, or benzene, averaged over ‘study’ period, binned by quintiles (pandas.qcut) (1,2,3,4,5)*  *US EPA conUS CMAQ 8-hour PM2.5 exposure estimate, averaged over 'study' period, binned by quintiles (pandas.qcut) (1,2,3,4,5)* |
| TotalEDInpatientVisits | *Total # ED/inpatient visits for respiratory issue(s) (defined by same ICD codes used to pull patients with asthma-like conditions) over ‘study’ period (0, 1, 2, 3, …)* |

*Abbreviations: ACS = American Community Survey; CDWH = Carolina Data Warehouse for Health; CMAQ = Community Multi-scale Air Quality modeling tool; DOT = Department of Transportation; ED = Emergency Department; IE = Institute for the Environment; PM25 = particulate matter ≤ 2.5 µm in diameter*

*The feature variables listed in the table are those for the patient-level tables, which include data on each patient for each year of available data (i.e., data on individual patients are represented as rows in the table). Similar feature variables are available for the visit-level tables, although the variables are sometimes treated differently. For example, PM_2.5_ exposures are expressed in relation to the 24-hour and two-week period before visits, not in relation to the one-year ‘study’ period, as was done for the patient-level tables. *Note that The FHIR PIT pipeline has been designed to integrate additional feature variables (e.g., US Department of Transportation roadway data, CDWH laboratory measures, US EPA CMAQ data on additional airborne pollutants); however, those data are not yet available via the ICEES OpenAPI, but will be released in the next version of the API.* Further information can be accessed via the [ICEES OpenAPI](https://icees.renci.org/apidocs).

^†^ Maximum age is 89 years, per HIPAA regulations

^††^The ICD codes for obesity and for the selection of patients with asthma-like conditions can be found in footnote two of the main manuscript.

^§^From first batch of CMAQ output, hourly estimates, 36-km (2010) or 12-km (2011) resolutions

**From second batch of CMAQ output, US Census tract resolution, 2002-2016 for PM2.5 and ozone

**Additional note: ICEES is currently restricted to patients with an asthma-like condition from UNC Health Care System.**

The following codes and parameters were used to identify patients with an ‘asthma-like’ phenotype:

**Diagnostic codes for asthma and asthma-like conditions** ICD9 493.% asthma ICD10 J45.% asthma ICD9 464.% croup ICD10 J05.% croup ICD9 496.% reactive airway ICD10 J44.% reactive airway ICD10 J66.% reactive airway ICD9 786.% cough ICD10 R05.% cough ICD9 481.% pneumonia ICD9 482.% pneumonia ICD9 483.% pneumonia ICD9 484.% pneumonia ICD9 485.% pneumonia ICD9 486.% pneumonia ICD10 J12.% pneumonia ICD10 J13.% pneumonia ICD10 J14.% pneumonia ICD10 J15.% pneumonia ICD10 J16.% pneumonia ICD10 J17.% pneumonia ICD10 J18.% pneumonia

**Tests and procedures for asthma and asthma-like conditions** CPT 94010 spirometry CPT 94070 multiple spirometry CPT 95070 methacholine challenge test CPT 94620 simple exercise stress test CPT

94621 complex exercise stress test CPT 31624 bronchoscopy CPT 94375 flow-volume loop CPT 94060 spirometry (pre/post bronchodilator test) CPT 94070 bronchospasm provocation CPT 95070 inhalation bronchial challenge CPT 94664 bronchodilator administration CPT 94620 pulmonary stress test CPT 95027 airborne allergen panel

**Medications prescribed for patients with asthma-like phenotype** MEDCTN prednisone MEDCTN fluticasone MEDCTN mometasone MEDCTN budesonide MEDCTN beclomethasone MEDCTN ciclesonide MEDCTN flunisolide MEDCTN albuterol MEDCTN metaproterenol MEDCTN diphenydramine MEDCTN fexofenadine MEDCTN cetirizine MEDCTN ipratropium MEDCTN salmeterol MEDCTN arformoterol MEDCTN formoterol MEDCTN indacaterol MEDCTN theophylline MEDCTN omalizumab MEDCTN mepolizumab
